# A gene set collection of human RBP target genes

**DOI:** 10.1038/s41597-026-07899-5

**Published:** 2026-08-29

**Authors:** Markus Glaß, Stefan Hüttelmaier

**Affiliations:** Institute of Molecular Medicine, Section for Molecular Cell Biology, Faculty of Medicine, Martin Luther University, Halle-Wittenberg, Germany

## Abstract

RNA binding proteins (RBPs) are key post-transcriptional regulators controlling every aspect of the RNA life cycle from synthesis to decay. We extracted RBP target genes from publicly available enhanced cross-linking and immuno-precipitation followed by sequencing (eCLIP-seq) data of 168 RBPs and assembled a gene set collection that can be used to examine gene lists for enriched RBP targets via functional enrichment analysis methods like over-representation analysis (ORA), gene set enrichment analysis (GSEA) or gene set variation analysis (GSVA).

## Background & Summary

RNA binding proteins (RBPs) comprise a class of proteins that bind to and interact with RNA molecules. The interactions between RBP and RNA can affect all aspects of the RNA’s life cycle, including synthesis, processing, post-transcriptional modification, localization, stability and degradation, in sum positioning RBPs as key regulators of RNA fate^1^.

RBP targets can be identified by techniques like RIP (RNA immunoprecipitation), CLIP (cross-linking and immunoprecipitation), or STAMP (Surveying Targets by APOBEC-Mediated Profiling), which capture RNA-protein interactions under native conditions, via UV crosslinking, or through fusion of an RBP to an RNA-editing enzyme, respectively^2,3^. Combined with high-throughput sequencing and bioinformatic analysis, these methods enable transcriptome-wide target identification. Several publicly available repositories and databases provide access to CLIP-seq data, including CLIPdb (http://111.198.139.65/), ENCORI (https://rnasysu.com/encori/), and the ENCODE project (https://www.encodeproject.org/)^4– 6^.

Functional enrichment analysis (FEA), also referred to as gene set analysis (GSA) methods encompass computational approaches used to determine whether certain properties of gene products appear more frequently than expected by chance. These properties can be multifaceted, e.g., the involvement in a particular biological pathway or disease, common chromosomal location or common targets of a regulatory molecule like an RBP. Several distinct FEA methods have been developed, including over-representation analysis (ORA), gene set enrichment analysis (GSEA) and gene set variation analysis (GSVA). For ORA, selected genes are tested for over-representation of certain properties among these genes. A background list of detected genes is essential for proper comparison to the list of selected genes^7^.

GSEA takes a different approach by considering the entire ranked list of genes (usually ranked by fold change between two experimental conditions) rather than just a subset. It calculates an enrichment score based on where genes from a particular gene set fall in the ranked list. This method does not require arbitrary cutoffs for defining significant genes^8^. GSVA, however, calculates enrichment scores for individual samples rather than comparing groups of samples. It uses a non-parametric approach that ranks genes within each sample and calculates sample-wise gene set enrichment scores as a function of genes inside and outside the gene set^9^. Thus, scores produced by GSVA can be interpreted as the degree to which a gene set is up- or down-regulated in each individual sample. Besides these three particularly popular methods, a multitude of further classes of FEA have been developed. An overview of methods and implementations can be found in^10^.

Despite the differences in the underlying statistics, all FEA methods rely on gene sets that provide the assignments between genes and the properties of their products. The Molecular Signatures Database (MSigDB)^11^ represents a comprehensive resource of gene sets applicable to FEA methods. The Human MSigDB is divided into ten collections, each representing gene sets focusing on different aspects, like the hallmarks, representing well-defined biological states or processes^12^ or the curated collection that contains gene sets derived from research papers and databases. Gene sets can be obtained from the MSigDB website (https://www.gsea-msigdb.org) as gene set (sub-)collections, stored in gmt format, a tab-delimited text file format, containing one gene set per line. While the MSigDB also contains a collection of regulatory target gene sets, this collection is restricted to microRNA and transcription factor targets. Thus, although the curated collection encompasses gene sets containing target genes of a few selected RBPs, e.g., BRCA1, IGF2BP1 and IGF2BP2, a comprehensive and focused collection of RBP targets is missing.

As a step toward closing this gap, we gathered high-confidence eCLIP data of 168 distinct RBPs provided by the ENCODE data portal (www.encodeproject.org). From the obtained peak files, we extracted genes associated with significantly enriched peaks and assembled a gene set collection representing associations of these 168 RBPs and the genes their target transcripts originated from. The collection is stored in the gmt file format and can directly be used with stand-alone programs like GSEA^8^ (www.gsea-msigdb.org/gsea/) or R-packages like clusterProfiler^13^ and GSVA^9^.

## Methods

### Data Collection

Relevant data sets were identified from the ENCODE data portal^6^ (www.encodeproject.org) by selecting the “eCLIP” assay on the “Experiment Search” page. Subsequently, irreproducible discovery rate (IDR) filtered peak files, containing significantly enriched eCLIP peaks reproducible between two independent biological replicates were obtained from the respective project pages in bed file format (downloaded October 2025). ENCODE-defined cutoffs for reproducible and significant peaks are ≥ 8-fold enrichment over size-matched input (geometric mean across 2 biological replicates), p-value ≤ 0.001 and irreproducible discovery rate (IDR) ≤ 0.01. The latter was ranked by the relative information content of a peak as metric to estimate the binding strength^14^. The full list of RBPs including ENCODE file identifiers is provided in table S1. Input data used for performing ORA and GSEA were obtained from supplemental data of open access publications. PUM2 PAR-CLIP data were obtained from Table S2 (NORAD WT CLIP targets) of^15^. Lists of alternatively spliced genes were taken from Table S1 of^16^. Differential gene expression data of THP-1 cells upon cGAMP stimulation were obtained from Table S1 (Sheet2) of^17^. Gene quantification data used for creating distinct background lists for use in ORA were obtained from ENCODE (downloaded April 2026): HCT-116 (ENCFF092ZUO, ENCFF906KQM), HeLa-S3 (ENCFF496CRF, ENCFF294GCU), Hep-G2 (ENCFF695GMA, ENCFF292KIL) and K-562 (ENCFF179CNW, ENCFF679BCM).

### Data Processing

Genes covered by peaks were obtained by applying the intersect command from the bedtools suite (v2.31.1)^18^ to obtain strand-specific overlaps between peaks and genes as annotated by ENSEMBL GRCh38 v114^19^ (obtained as gff3 file from https://ftp.ensembl.org/pub/release-114/gff3/homo_sapiens/). Thus, peaks not overlapping genes annotated by this reference were not considered further. If peaks overlapped multiple genes located on the same strand, all respective intersections were extracted. From the resulting intersection files, gene names were extracted and a gmt file was created that contains one gene set for each RBP in each available cell line.

## Data Record

**Fig. 1:**
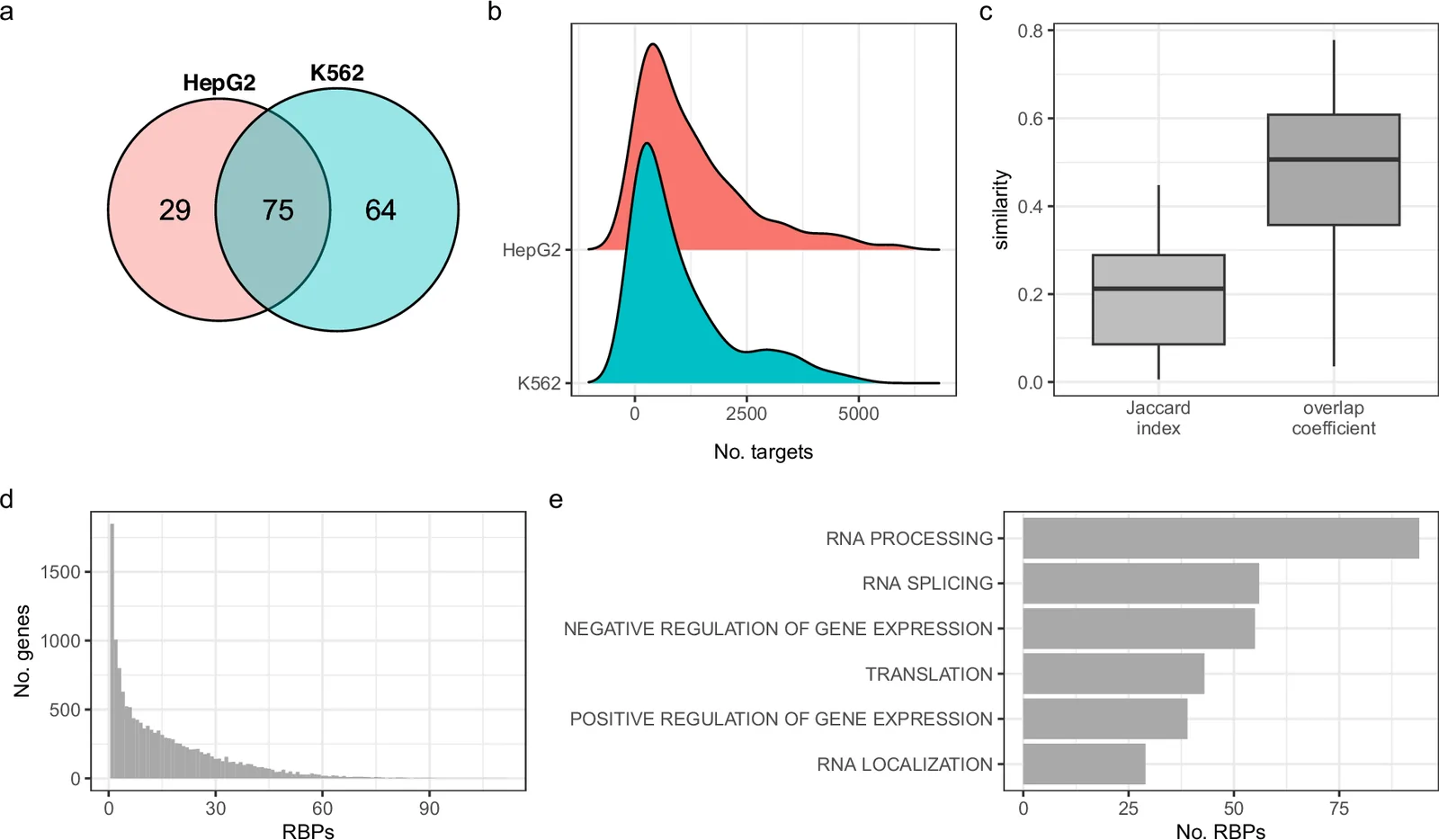
Overview of the RBP gene set collection. (**a**) Distribution of eCLIP experiments among the two applied cell lines Hep-G2 and K-562. (**b**) Distribution of derived target gene numbers among the two distinct cell lines. (**c**) Jaccard-indexes and overlap coefficients of those 75 eCLIP-experiments performed for the same RBP in both cell lines. (**d**) Number of target genes shared by distinct RBPs. (**e**) Selected Gene Ontology-derived biological processes in which the RBPs from the gene set collection are involved.

The gene set collection is comprised of 243 gene sets containing putative target genes of 168 distinct RBPs determined from either hepatoblastoma-derived Hep-G2 or chronic myeloid leukemia-derived K-562 cells or both (Fig.1a). 150 of these 168 RBPs (89.3 %) were present in the curated census of 1,542 human RBPs compiled by Gerstberger *et al*.^20^, representing approximately 10 % of that reference set. In general, more RBP target genes were derived from eCLIP-data originating from Hep-G2 cells than from K-562 cells (median target gene number 860.5 vs. 596; Fig. 1b). Overlap coefficients and Jaccard indices calculated between target gene lists of the same RBP, derived from experiments conducted in either Hep-G2 or K-562 cells, yielded median values of 0.51 and 0.21, respectively, indicating that while approximately half of the target genes identified in the cell line with less targets were also identified in the other, the considerably lower Jaccard index reflects the high variability in target gene list sizes between cell lines, since this metric penalizes genes detected in only one of the two lists (Fig. 1c). This might reflect differences in the technical feasibility of eCLIP in dependence of the respective cell models as well as differences in the overall expression profiles of the distinct cell lines. In total, 14,411 distinct target genes comprised the gene set collection. Of those, 1848 genes were uniquely associated with one RBP, whereas the remaining target genes were shared by at least two distinct RBPs (Fig. 1d). Biological functions of the RBPs included in the gene set collection covered processes like splicing, gene expression regulation, translation and localization (Fig. 1e).

The gene set collection is available as gmt file at Zenodo (https://doi.org/10.5281/zenodo.17296967^21^) and from GitHub (github.com/HuettelmaierLab/RBP_Gene_Set_Collection). Each line of the gmt file comprises names of genes cross-linked to a specific RBP in either Hep-G2 or K-562 cells. The name field, i.e., the first entry of each line, specifies the RBP and cell line, the second field (description) represents the ENCODE identifier of the peak file of which the target genes were obtained from.

## Technical Validation

To test usability and plausibility of our gene set collection, we used it in conjunction with different R-packages to perform ORA, GSEA and GSVA using publicly available gene lists as input.

**Fig. 2:**
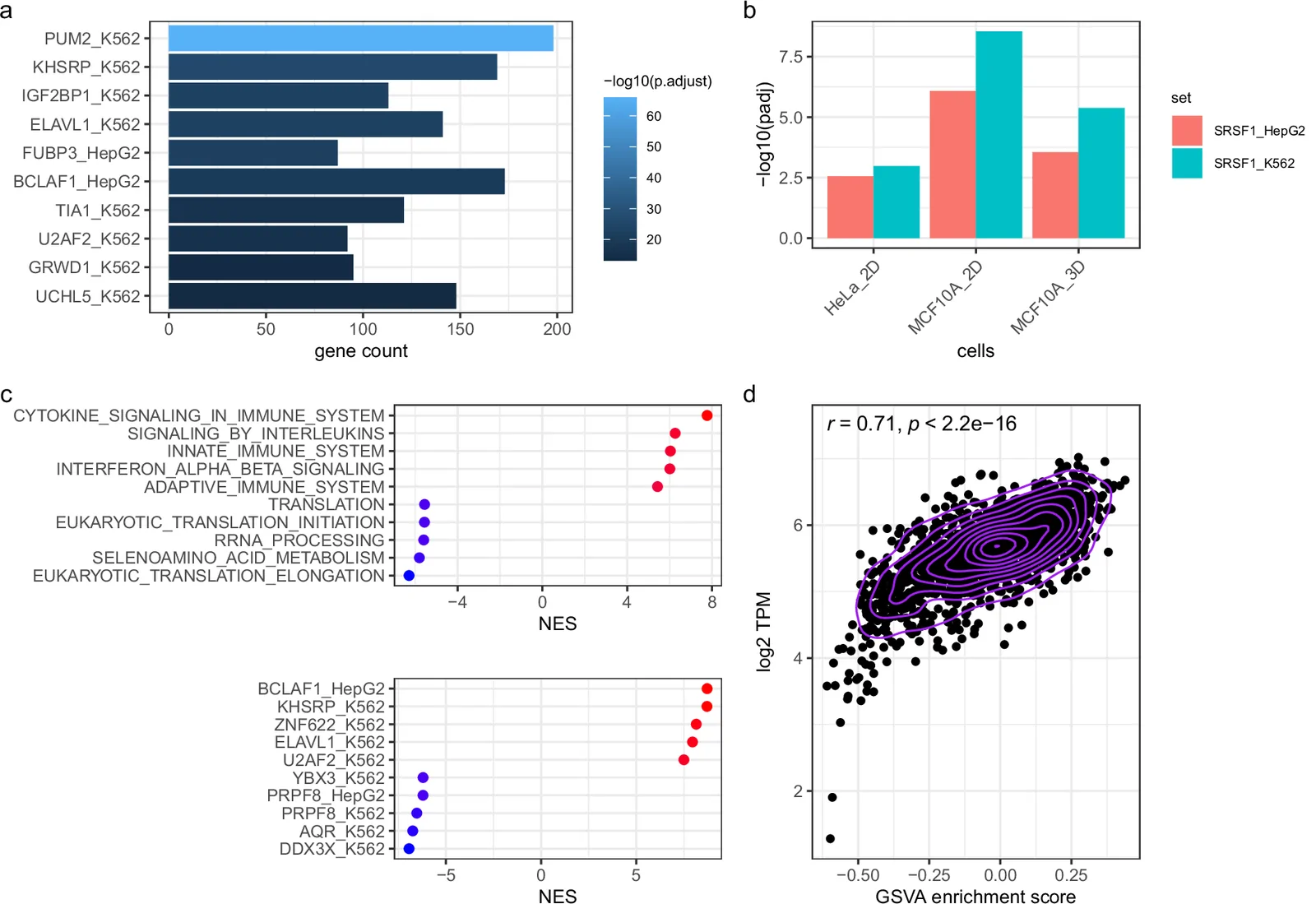
Functional enrichment analysis (FEA) results using the proposed RBP target gene set collection. (**a**) Top 10 most significantly enriched RBP gene sets using the PUM2 PAR-CLIP-derived genes as input gene list for an ORA. (**b**) ORA-derived FDR adjusted p-values regarding enrichment of SRSF1-related gene sets using lists of genes alternatively spliced upon SRSF1 overexpression in the indicated cell models as input. (**c**) Normalized enrichment scores (NES) of the top 5 positively and negatively enriched Reactome (upper panel) and RBP gene sets (lower panel) upon GSEA using log2 fold changes of gene expression upon cGAMP stimulation. All displayed gene sets were significantly enriched (FDR adjusted p-values  < 0.05). (**d**) GSVA enrichment scores of ELAVL1 target genes and ELAVL1 RNA expression from 1404 distinct CCLE cell line data sets. r: Pearson correlation coefficient. Purple lines represent density curves.

For testing plausibility, we first performed an ORA using a gene list derived from a PAR-CLIP^22^ experiment using endogenous Pumilio 2 (PUM2) protein for pull-down in colon cancer-derived HCT-116 cells. The gene list obtained from supplemental data of Lee *et al*.^15^ contained 370 distinct genes. In contrast, the respective PUM2 eCLIP gene set, incorporated in our collection, contained almost 3000 target genes. Of those, 198 were also contained in the PAR-CLIP list. Despite this large difference in target gene numbers, ORA results, obtained by using the enricher function of the clusterProfiler R-package^13^, utilizing the entirety of genes included in the gene set collection as background (n = 14,411), revealed PUM2 targets to be the most significantly enriched gene set, containing the most genes of the input PAR-CLIP list (Fig. 2a). Thus, PUM2 target genes derived from an orthogonal method could be identified as being enriched for PUM2 targets using our eCLIP-derived RBP target gene set collection.

Since pure binding, as determined by methods like PAR-/eCLIP, not necessarily reflects functional consequences, we next tested if genes associated with functions of a certain RBP could be used to refer to this RBP as well. Therefore, we used lists of genes identified to be alternatively spliced upon over-expression of the splicing factor SRSF1 for ORAs. These gene lists were generated by analyzing RNA-seq data of control cells in comparison to cells over-expressing SRSF1 for alternative splicing events by Anczuk *et al*. Experiments were performed in three different cell models, 2D-cultured cervical cancer-derived HeLa cells, 2D-cultured breast cancer-derived MCF-10A cells as well as 3D-cultured MCF-10A cells^16^. Although not found among the most significantly enriched gene sets, SRSF1 target gene sets were significantly enriched (FDR adjusted p-value ≤ 0.05) in ORA results using the detected alternatively spliced genes from all three cell models as input (Fig. 2b). Of the 332 alternatively spliced genes detected in HeLa cells, 35 (SRSF1_K562) and 32 (SRSF1_HepG2) matches were found. Likewise, 58 and 50 genes out of 403 matched the MCF-10A, 2D-derived alternatively spliced genes as well as 63 and 54 out of 517 genes detected to be alternatively spliced in 3D-cultured MCF-10A cells.

To test the gene set collection for applicability with GSEA, we obtained a list of gene expression fold changes between control and cyclic GMP-AMP (cGAMP)-stimulated monocytic THP-1 cells. cGAMP was used as a surrogate for viral infection by acting as an agonist of the immune adaptor stimulator of interferon genes (STING). The authors of this study performed further high-throughput sequencing assays, including PAR-CLIP to conclude that upon cGAMP stimulation the RBP ELAVL1 is activated by post-translational modification leading to increased stabilization of its target transcripts^17^. GSEA performed using R/clusterProfiler^13^ on the MSigDB curated sub-collection containing Reactome pathways^23^, revealed several gene sets related to the innate immune response as being the strongest positively enriched gene sets. Gene sets showing the strongest negative enrichments were connected to mRNA translation (Fig 2c). GSEA performed with the RBP collection revealed targets of immune system-related RBPs, including ELAVL1 as being the most strongly positively enriched sets^24–27^. Furthermore, the gene sets showing the strongest negative enrichment were associated with RBPs related to the immune system as well (DDX3X, AQR), but also associated with RBPs known to regulate RNA translation (DDX3X, YBX3) ^28–31^. GSEA using the stand-alone GSEA application^8^ in pre-ranked mode and the classic scoring scheme yielded highly similar results (data not shown).

Finally, we tested our gene set collection in conjunction with GSVA. We obtained RNA expression values of 1404 distinct cell line models available provided by the cancer cell line encyclopedia via the R-package ExperimentHub^32,33^ and used this dataset as input for the gsva function of the GSVA R-package^9^. After obtaining enrichment scores of all RBP gene sets, we calculated the correlation between ELAVL1 RNA expression and GSVA enrichment scores of ELAVL1 target genes. This revealed a strong positive correlation suggesting that ELAVL1 exerts a promoting impact on its RNA targets supporting its reported stabilizing role^17^ (Fig 2d).

## Usage Notes

**Fig. 3:**
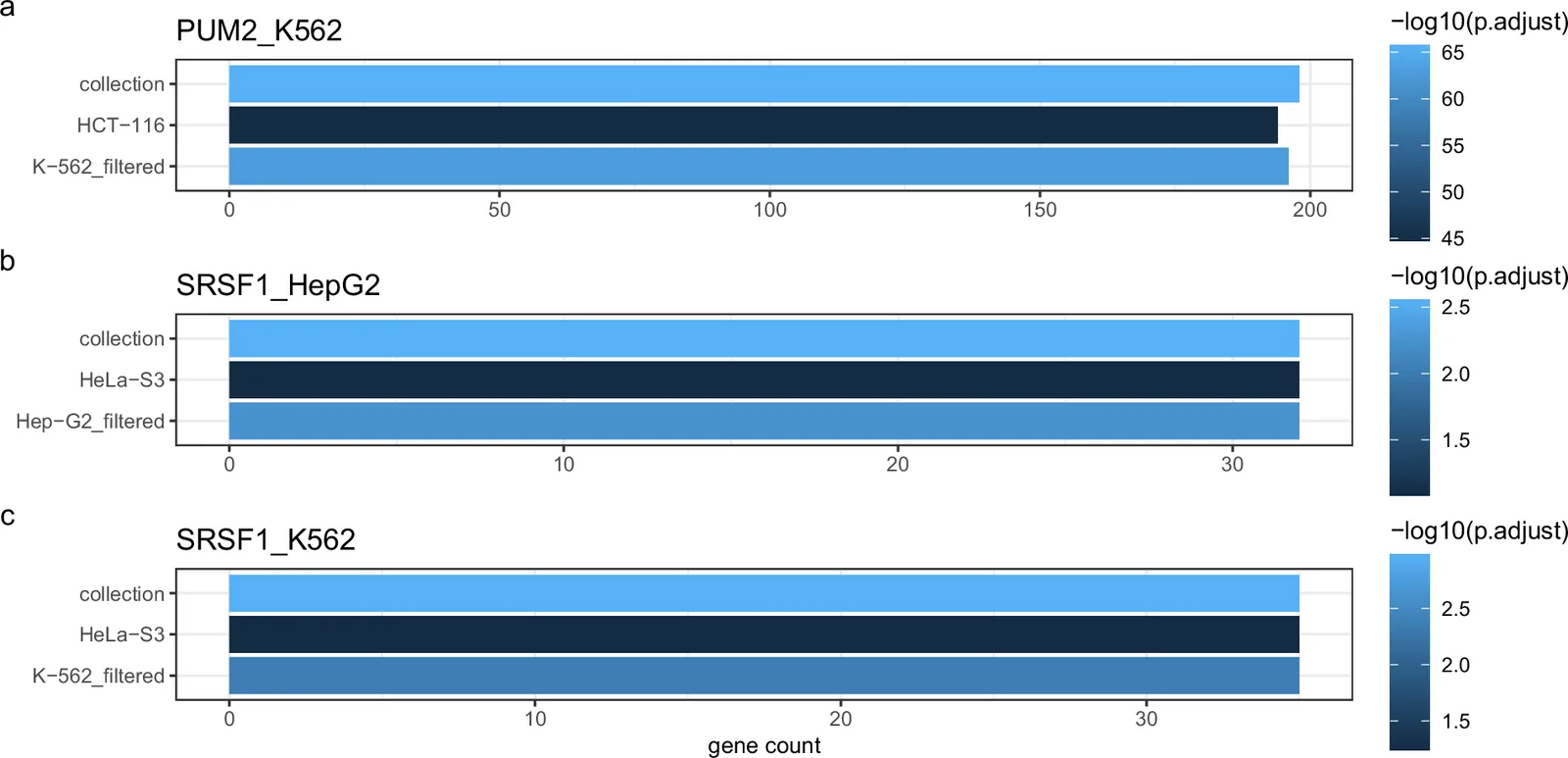
Influence of different background gene lists on ORA results. (**a**) ORA result for the PUM2 eCLIP gene set derived from K-562 cells using different background and input gene lists: collection - all target genes from the gene set collection as background, all genes associated with PAR-CLIP peaks as input; HCT-116 - only genes expressed in HCT-116 cells (avg. TPM ≥ 1) and contained in the gene set collection used as background, all genes associated with PAR-CLIP peaks as input; K-562_filtered - only K-562 derived gene sets used, target genes contained in these sets used as background, only those PAR-CLIP targets used as input, that are expressed in K-562 cells (avg. TPM ≥ 1). (**b,** **c**) ORA results for the SRSF1 eCLIP gene sets derived from Hep-G2 (b) or K-562 (**c**) cells using different background and input gene lists: collection - all target genes from the gene set collection as background, all differentially spliced genes as input; HeLa-S3 - only genes expressed in HeLa-S3 cells (avg. TPM ≥ 1) and contained in the gene set collection used as background, all genes associated with PAR-CLIP peaks as input; Hep-G2_filtered/K-562_filtered - only Hep-G2/K-562 derived gene sets used, target genes contained in these sets used as background, only alternatively spliced genes used as input, that are expressed in Hep-G2/K-562 cells (avg. TPM ≥ 1).

The ORA analyses presented in Fig. 2 used clusterProfiler’s default background setting, in which all unique gene symbols from the applied gene set collection (14,411 predominantly protein-coding genes for the RBP collection) serve as background. As recommended by Ziemann *et al*.^7^, however, the background should be restricted to detected genes. To assess this impact, we repeated the ORAs under three parameter set-ups: (I) all genes from the gene set collection as background (default); (II) only genes expressed at reasonable levels (avg. TPM ≥ 1) in the relevant cell lines, based on bulk RNA-seq data from the ENCODE portal; (III) only gene sets from eCLIP experiments in a single cell line (K-562 or Hep-G2), with background restricted to RBP targets in those sets and input restricted to genes expressed (avg. TPM ≥ 1) in the same cell line. Note that clusterProfiler’s enricher function uses the intersection of the gene set collection and the specified background, precluding the use of genes absent from the collection. For the PUM2 PAR-CLIP data, restricting the background to HCT-116-expressed genes (n = 13,892) marginally reduced target gene matches from 198 to 194; using only K-562-derived gene sets and filtering unexpressed PAR-CLIP targets yielded 196 matches with the K-562 PUM2 eCLIP gene set. Across all three set-ups, the PUM2 gene set consistently showed the highest overlap and lowest adjusted p-values (Fig. 3a). Likewise, ORA results for genes alternatively spliced upon SRSF1 overexpression in HeLa cells were only modestly affected by background restriction to HeLa-, Hep-G2-, or K-562-expressed genes (Fig. 3b/c).

**Fig. 4:**
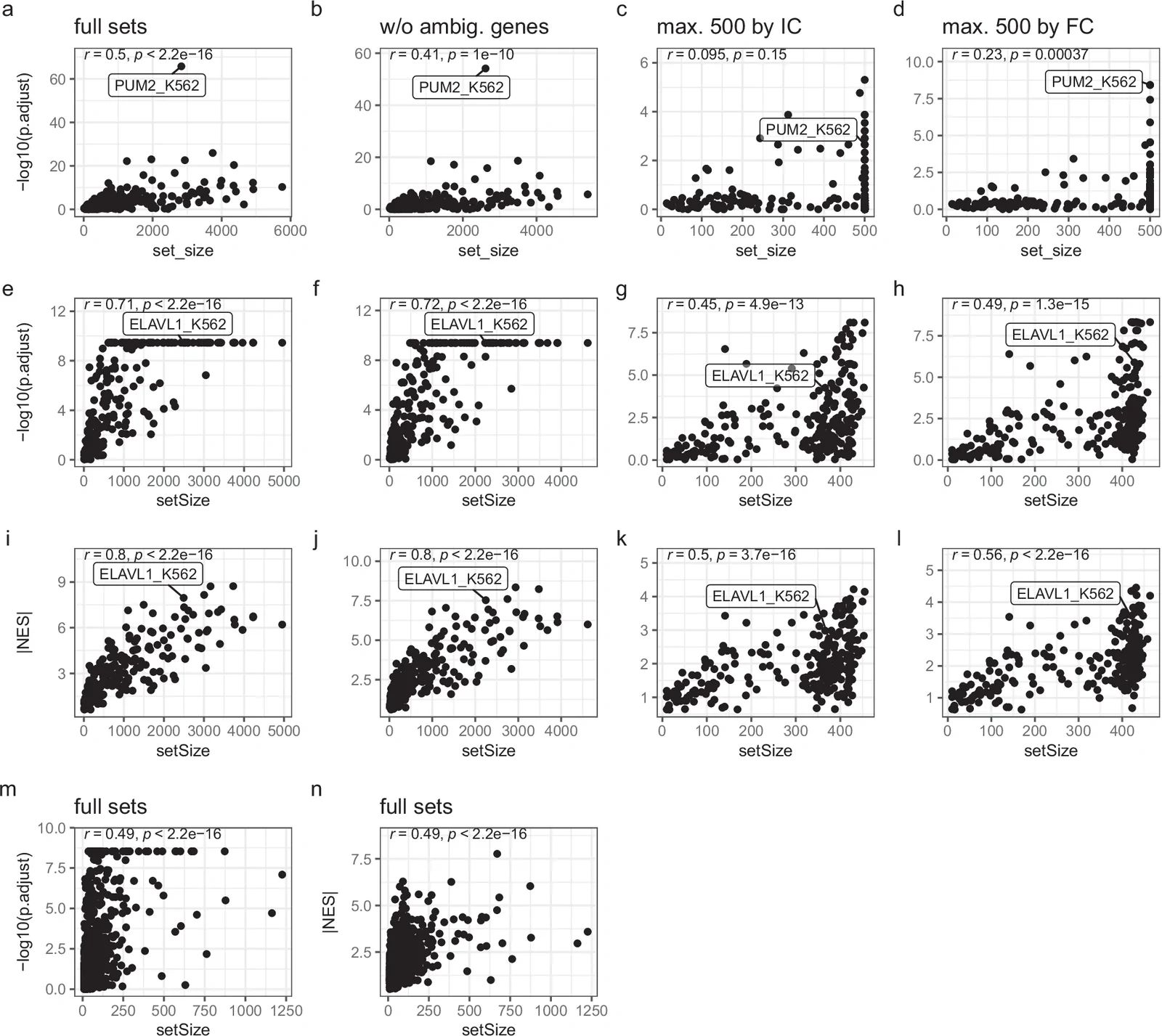
Influence of gene set size on FEA results. ORA using PUM2 PAR-CLIP targets (1st row) and GSEA using log2 fold changes of gene expression upon cGAMP stimulation (2nd to 4th row) using distinct RBP gene set collection variants. (**a,e,i**) No restriction of gene set size. (**b,f,j**) Genes found to be targets of more than 1/3 of all RBPs removed. (**c,g,k**) Gene sets restricted to the 500 genes with highest information content (IC). (**d,h,l**) Gene sets restricted to the 500 genes with highest average fold change (FC) of eCLIP peaks over input. (**m,n**) GSEA using log2 fold changes of gene expression upon cGAMP stimulation using the MSigDB C2 collection Reactome sub set. r: Pearson correlation coefficient.

Besides the assumptions of equal importance and independence of the genes, another known limitation of ORA is its sensitivity to gene set size, since larger sets increase the probability of finding hits by chance. Our RBP gene set collection ranged from 5 to 5,757 genes per set, and we observed significant positive correlations between gene set size and adjusted p-values in both ORA and GSEA results (Fig. 2a/c). GSEA normalized enrichment scores (NES), though size-adjusted, also correlated strongly with set sizes — a trend likewise seen with MSigDB Reactome gene sets (Fig. 4a/e/i/m/n). To test whether these size dependencies could be mitigated, we constructed three modified collections: (I) genes targeted by more than one third of all RBPs (n = 56) were removed from individual gene sets and consolidated into an “ambiguous_targets” set, preserving the total gene count (389 genes); (II) gene sets were capped at 500 genes, retaining those genes with the highest information content (defined as the negative logarithm of the fraction of RBPs targeting a given gene among all 168 RBPs); (III) gene sets were similarly capped at 500, retaining genes with the highest average eCLIP peak fold change over input. Relocating promiscuous targets modestly affected ORA and GSEA results (Fig. 4b/f/j), while capping gene set sizes substantially reduced size-p-value correlations. However, this also reduced adjusted p-values and NES for the expected gene sets, limiting practical utility for the use-cases presented here (Fig. 4c/d/g/h/k/l). RBPs associated with very large target gene sets, like BCLAF1, generally rank quite high in the tests performed for this study. This represents a methodological bias of the FEA methods, but also the fact, that promiscuous RBPs with thousands of targets may appear to be enriched just because their binding repertoire is so broad. Their target sets might overlap any biologically coherent gene list simply by virtue of transcriptome-wide coverage, rather than specific regulatory relevance. Exclusion of these RBPs could alleviate this bias and most FEA software tools offer parameters to remove sets exceeding defined set sizes.

## Supplementary information


Supplementary Information


## Data Availability

The most recent version of the gene set collection is available in gmt file format from github.com/HuettelmaierLab/RBP_Gene_Set_Collection. The version described in this manuscript is available at Zenodo (https://doi.org/10.5281/zenodo.17296967).

## Code availability

R-scripts used to create the gene set collection, including wrapping code of the bedtools commands, as well as R-scripts and gene lists for performing the FEAs described in the Technical Validation section are available at Zenodo (https://doi.org/10.5281/zenodo.17296967).
